# Direct Determination of 3D Distribution of Elemental Composition in Single Semiconductor Nanoislands by Scanning Auger Microscopy

**DOI:** 10.1186/s11671-016-1308-x

**Published:** 2016-02-24

**Authors:** Semyon S. Ponomaryov, Volodymyr O. Yukhymchuk, Peter M. Lytvyn, Mykhailo Ya Valakh

**Affiliations:** Institute of Semiconductor Physics, NASU, Pr. Nauky 41, Kyiv, 03028 Ukraine

**Keywords:** Single quantum dots, 3D composition distribution, Opened and capped nanostructures, Scanning Auger microscopy, Gigantic interdiffusion, 81.07.Ta, 68.65.Hb, 68.37.Xy

## Abstract

An application of scanning Auger microscopy with ion etching technique and effective compensation of thermal drift of the surface analyzed area is proposed for direct local study of composition distribution in the bulk of single nanoislands. For Ge_x_Si_1 − x_-nanoislands obtained by MBE of Ge on Si-substrate gigantic interdiffusion mixing takes place both in the open and capped nanostructures. Lateral distributions of the elemental composition as well as concentration-depth profiles were recorded. 3D distribution of the elemental composition in the d-cluster bulk was obtained using the interpolation approach by lateral composition distributions in its several cross sections and concentration-depth profile. It was shown that there is a germanium core in the nanoislands of both nanostructure types, which even penetrates the substrate. In studied nanostructures maximal Ge content in the nanoislands may reach about 40 at.%.

## Background

Quantum dots (QDs) and other nanoobjects, where electrons are subjected to dimensional confinement, attract considerable interest of researchers and technologists because of their anticipated application in optoelectronics and quantum informatics [1–5]. These structures may be used for creation of LEDs promising for optic systems, as well as for communications and laser diodes with high electroluminescence intensity [6–9]. Self-induced growth by heteroepitaxy under Stransky-Krastanov growth mode is one of the most attractive ways of creating such objects due to a number of technological reasons [10]. This way allows producing huge homogeneous arrays of QDs relatively easy and fast [11, 12]. In this case, nanoislands are formed on the growing film because it accumulates high mechanical stresses due to lattice mismatch between film and substrate [13, 14]. In the case of Ge epitaxy on Si-substrate, this mismatch equals 4 %. The 3D growth mode instead of planar film growth is more energy-saving as the nanoislands are not subjected to lateral constraints, partially relax and further do not accumulate mechanical stresses intensely [15].

However, the film in the process of its growth changes not only its morphology, but in general case also its composition [16]. Actually, equilibrium state of Ge/Si-systems is their homogeneous solution in each other (Si and Ge form continuous series of solid solutions), and interdiffusion is the only way to reach it [17–19]. In other words, formation of the nanoislands is not the ultimate equilibrium state which the system tends to, but rather a step in its direction [20]. High film growth rates, small deposition periods (several minutes), and low deposition temperatures (450 ÷ 550 °С) are the factors limiting diffusion kinetics. Diffusion may be ignored under these conditions for the whole film-substrate system, but it should be considered in the nanosize system areas with high mechanical stresses, huge gradients of concentration, and high-rate ways of surface transport of the substance. Wetting layer with growing nanoislands is the system area in question. The above statement is supported by the numerous experimental data showing that composition of the nanoislands and the wetting layer essentially differs from the composition of the deposited material [17–19, 21, 22].

Lateral size of the typical semiconductor nanoislands varies within the range of 3–80 nm; therefore, the electrons there are subjected to quantum confinement specifying, for example, photoluminescence emission [23]. But the last one may be even more seriously depends on chemical composition distribution [24]. Therefore, knowledge of the real distribution of composition in a single nanoisland is extremely important for applications. On the one hand, it may help understand the nature of photoluminescence in structures with the nanoislands and find the way of increasing its yield [25], and on the other, clarify in many ways unclear issues related to details of the QDs formation mechanism and a type of their evolution while growing.

Scanning probe microscopy techniques are widely and successfully employed for studying geometrical parameters and morphological peculiarities of nanostructures. As for studying the nanoclusters composition, the nonlocal techniques are mostly used [26]: the Auger electron spectroscopy, X-ray photoelectron spectroscopy, X-ray diffraction [27], Raman spectroscopy, and others [28, 29]. They only provide data about average content of Ge and Si in the nanoislands. Transmission electron microscopy (TEM) with various analytical equipment is used in the most cases for determination of the nanoislands local composition [26, 30]. It is used to study the local composition of both open and buried nanostructures. However, the TEM requires rather complicated procedures for specimen preparation. In this case, the prepared foils have only small areas suitable for study, which does not provide reliable statistics of the obtained results. It is also noteworthy that interpretation of the data got the TEM is nontrivial due to averaging composition along *Z* axis and position uncertainty of the studied foil surface plane (cross section plane) relative to the axis of the nanoislands.

Currently, there is a lot of experimental data on the distribution of local composition in the bulk of an individual GeSi-nanoisland. However, they are in quite contradictory character. Thus, in one study [31], it was shown that nanoisland has Ge-core, and the other [32] presents experimental data indicating that the nanoisland surface layers are enriched by Ge. In our view, the contradictory nature of the available data is due to the fact that the methods used to obtain them are not direct. Therefore, they require interpretation, which is based on a priori model ideas that describe the object under study and the scheme of measuring experiment. Apparently, contradictions arise from the fact that these ideas are not always adequate.

The authors of this work used scanning Auger microscopy (SAM) technique, which is a combination of Auger electron spectroscopy and high-resolution scanning microscopy (SEM), to study distribution of the local composition in the GeSi/Si-QDs as an example of semiconductor nanoislands. This technique is ideal for solving the mentioned problem, since the size of its analyzed area is about 3–5 nm in the lateral plane and about 1 nm depthward [33]. There is a crucial factor for successful application of SAM for local studies of the composition of single nanoislands, which is an effective control of thermal drift of the analyzed site during rather extended procedure of the Auger spectra recording by regular electronic correction of the nanoisland position on the SEM image of the heterostructure surface area under study. It is noteworthy that SAM technique was for the first time applied to study the local composition of GeSi/Si-nanoislands in the pioneering works by Nikolichev and Maximov with the colleagues [34, 35]. However, the available equipment allowed analyzing rather large nanoobjects (from 125 to 600 nm in diameter), which is of low practical interest.

## Methods

The nanoislands studied in this work were grown in the molecular beam epitaxy setup “BALZERS” under residual atmosphere pressure of 10^−7^–10^−8^ Pa. The specimens of the two series were studied.

Series А consisted of two specimens. The specimen А 1 was produced by depositing Ge on Si (001) substrate with rate *v*_dep_ = 0.07 Å/s at the temperature *T*_grow_ = 700 °С. The nominal thickness of the germanium layer was 8.7 ML. Right after completion of the nanoislands growth, the structures began to cool down at the rate of 1 °С/s. The second specimen А 2 of this series was grown under the same conditions. In this case, Ge was deposited on the buffer layer Si_0.9_Ge_0.1_ with 10 nm thickness, while the nominal thickness of the germanium layer was 8 ML.

The second series В also consisted of two specimens. One of them is В 1 specimen with an array of nanoislands grown at the temperature *T*_grow_ = 700 °С by depositing 11 ML Ge on the Si_0.9_Ge_0.1_ buffer layer 10-nm thick, with subsequent silicon capping. Initial stage of the nanoislands capping was performed at relatively low temperature of 300 °С. When the islands were completely covered with the Si layer, the epitaxy temperature increased up to 500 °С with further growth of the silicon layer to 80 nm thick. В 2 specimen is a complex heterostructure containing QDs with previously an unknown geometry and composition of layers. The specimen was used to demonstrate the ability to determine the composition and thickness of the layers of unknown heterostructure with the developed technique.

Studies were performed on the Auger microprobe JAMP 9500F (JEOL), with 3 nm resolution in the secondary electron image mode. The microprobe was equipped with sensitive hemispheric Auger spectrometer with energy resolution ΔE/E from 0.05 to 0.6 % and an ion etching gun for layer-by-layer analysis with diameter of Ar^+^ ion beam 120 μm, able to move by raster 1 × 1 mm. Variation range of the beam Ar^+^ ion energy is from 0.01 to 4 keV, while minimal beam current is 2 μA with 3 keV. For the analysis, the analytical Auger peaks of Si_KLL_ and Ge_LMM_ with energy 1619 and 1147 eV, respectively, were used. In the case of registration of Si-distribution, Auger map was used Si_LVV_ peak with energy 92 eV, since it is more intense. Its use has significantly reduced the Auger maps registration time.

## Results and Discussion

The main goal of the study was, using scanning Auger microscopy technique, to determine the elemental distribution types in a single nanoisland and wetting layer, to estimate Si and Ge interdiffusion scale in various elements of Ge/Si-heterostructure, and to clarify surface diffusion role in nanoisland structure formation.

### Ge and Si Lateral Distribution in Single Nanoislands

To start with, let us consider the problem on obtaining image of the surface area containing GeSi QDs by the scanning Auger electron microscopy. We will call it “Auger map”. The goal of obtaining this image is to establish lateral distribution of Si and Ge elements on the specimen surface with nanoislands. Since lateral size of the studied structures is in the range of 30 to 70 nm, mapping should be performed at high magnification and high resolution in Auger and secondary electron image modes, which requires overcoming a number of difficulties.

First of all, it is the thermal drift that should be controlled when working at the magnifications above ×20,000. Long period of the Auger maps and Auger spectra recording is a consequence of reaching highest possible spatial resolution due to at most pointed probe. Small diameter of the electron probe is determined by a low current of the probe and thus a weak signal, which needs to be accumulated by making up to seven passes on the same raster of the Auger electron map. Long period of the Auger map recording (from 2 to 20 h) also contributes to the thermal drift.

In order to solve the above problems, a procedure of electronic correction for the thermal drift was developed. The idea of the procedure is successively to compare shifted current image with the reference image of the studied GeSi QDs array in time intervals (<10 s), for which the array does not shift noticeably (>5 nm), and to match these images. Accuracy of such matching can be evaluated as 5–10 nm. The ultimate lateral resolution of the scanning Auger microscopy images is determined by this value. Therefore, each raster line of the Auger map image was recorded for less than 10 s. Electronic correction of the probe position described above was performed after each raster line.

**Fig. 1:**
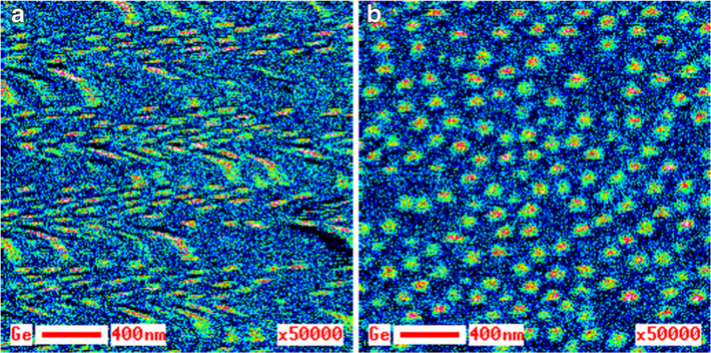
Auger map of Ge on the А 1 specimen surface. Auger map of Ge distribution on the surface of the specimen А 1 containing GeSi QDs without electronic correction of the thermal drift (**a**) and with it (**b**)

Solution of the thermal drift problem plays an exceptionally important role, which is easy to see while comparing the two Auger maps of Ge distribution presented in Fig. 1a, b. Both maps are taken from the same area of the specimen surface, but in different modes. The first map was taken at magnification ×50,000. Due to low intensity of Ge peak in the Auger spectrum, the recording time was 2 h. In this case, no electronic correction of the thermal drift was conducted. The second map was also taken at magnification ×50,000, but with electronic correction of the thermal drift. When recording the Auger map in Fig. 1b, the time was distributed between correction procedure (90 % of the total time period), and signal accumulation (10 % of the total time period). The conducted correction managed to fully remove shift of the analyzed site image during analysis, while in the first case, it was in place. In Fig. 1, as further for the Auger maps, hot color corresponds to a high content of the analyzed element, while a cold color—to its low content.

It is important to answer the question on distribution of the elemental composition on the surface of the specimen containing GeSi QDs. By using the developed procedure of the thermal drift correction, it is possible to obtain the Auger maps of high spatial resolution for Si and Ge lateral distribution and to answer this question. By comparing the obtained results with the SEM image of the same specimen surface area, it becomes possible to determine correlation between elemental composition distribution and morphological features of QDs.

**Fig. 2:**
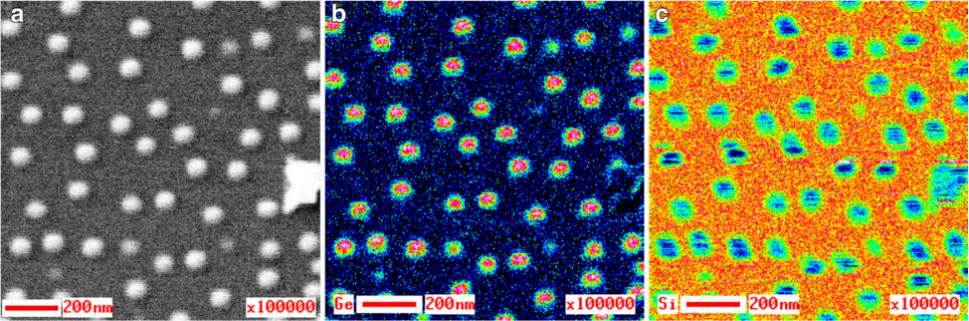
SEM image and the Auger electron maps of the A 1 specimen surface. SEM image of the analyzed surface area of the specimen А 1 (**а**) and the Auger electron maps corresponding to this area for Ge (**b**) and Si (**c**) distributions, respectively

Figure 2 presents SEM image of the surface area of the specimen А 1 (a), as well as the Auger electron maps corresponding to this area for Ge (b) and Si (с). As it is seen from the SEM image, lateral size of GeSi QDs is from 40 to 70 nm. Structures appearing on the Ge Auger map have sharp contours and are easily comparable with the nanoislands on the SEM image of the same area of the specimen surface. It is seen from Fig. 2b that a maximal quantity of Ge is in the central part of GeSi QDs, and it decreases by its periphery, while the minimal quantity is in the wetting layer. This experimental fact is confirmed by the Auger map of Si distribution, which in its type is complementary to the Ge Auger map. Minimal Si quantity corresponds to the surface areas with GeSi QDs, while maximal Si quantity is in the wetting layer.

Therefore, analysis of the Si and Ge Auger maps supports a conclusion that GeSi QDs have germanium core surrounded by the silicon shell [36].

It is evident that such distribution of the elemental composition may appear only due to intensive diffusion of Si from the substrate and Ge into the substrate. Silicon distribution in Fig. 2c testifies to the fact that it diffuses from the substrate not only to the GeSi QDs shell, but also to the wetting layer surface.

### Ge and Si Concentration Depth Profiles in Single Nanoislands

The Auger image allows obtaining the map of the element distribution on the specimen surface with high spatial resolution. However, it is equally important to learn how the composition is distributed depthward in both GeSi QDs and the wetting layer, i.e., to learn concentration depth profiles. In this case, we are talking about the layer-by-layer analysis: (i) local determination of the elemental composition in the specimen surface sites of interest (wetting layer, pyramid nanoisland or р-cluster, dome nanoisland or d-cluster) [37, 38], (ii) successive removal of surface layers of preset thickness by the ion etching and repeated analysis in the same points. As a result, we obtain concentration depth profiles—distribution of the composition depthward in the specimen in the surface sites of interest.

If before with the Auger image, we reduced the accumulation time to the one raster line increasing the number of repeated registrations of this line in order to correct the thermal drift, then in case of concentration depth profile, we reduced the spectrum accumulation time to 10 s. Then the thermal drift was compensated, and the same spectrum was recorded once again. The obtained spectra were summed. Their quantity was determined by obtaining acceptable signal/noise ratio.

It might be well to point that in removing the surface layer of the preset thickness by ion etching, it is necessary to take into consideration and minimize various effects of the surface modification, such as mixing, selective etching, and so on. In this case, soft ion etching modes (energy of Ar^+^ ions was 1 keV, ion beam current 5.7 × 10^−7^ A), short-length etching periods (10 s), and long periods of the surface relaxation (>20 min)) should be used. Diameter of the ion beam moving on the raster of 1 × 1 mm in size was 120 μm.

In order to obtain the concentration depth profile, it is necessary to know an average rate of the specimen surface etching. It is determined as follows: lateral size of the p-cluster base—*l* was determined from the SEM images of the specimen surface. As it is known from the STM data [39], р-cluster has regular-shaped faceting of the pyramid, where the lateral facet of the pyramid is a plane (105) inclined at an angle *α* of 10.9° to the base plane (001). It is easy to find the p-cluster height *h* from the obtained value of the lateral size:

$$ h=\frac{l\cdot \tan \alpha }{2}. $$

**Fig. 3:**
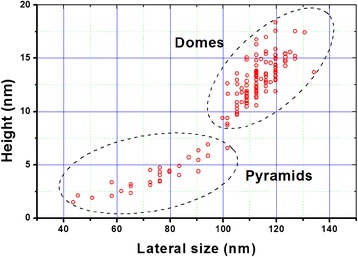
P- and d-nanoislands size distribution, registered with AFM technique on А 1 sample. Their average height is 4 and 14 nm, respectively

The p-cluster height *h* obtained by this way is in a good agreement with its determination by AFM technique (see Fig. 3).

The above described procedure of the layer-by-layer analysis was used to get concentration Auger profiles of C, O, Ge, and Si depth distributions in a single p-cluster and a wetting layer nearby. If it is remembered that p-cluster sits on the wetting layer, then its etching time may be determined as *t*_*p* + wl_ = *t*_*p*_ + *t*_wl_, where *t*_*p*_ is p-cluster etching time from its top to its bottom, *t*_wl_—wetting layer etching time. From all has been said, it follows that an average etching rate of p-cluster 〈*v*_*p*_〉 is determined by the formula:

$$ \left\langle {v}_p\right\rangle =\frac{h}{t_{p+\mathrm{w}\mathrm{l}}-{t}_{\mathrm{wl}}}=\frac{l\cdot \tan \alpha }{2\left({t}_{p+\mathrm{w}\mathrm{l}}-{t}_{\mathrm{wl}}\right)}. $$

By finding the average rate of the specimen surface ion etching (≈0.1 nm/s) and considering the time spent for etching in a cycle of the depth profile (10 s), one may estimate thickness of the removed layer per a cycle as ≈1 nm.

For obtaining the concentration depth profiles, it is also necessary to pay attention to the correct quantitative element analysis. This problem was solved by calibration of the relative sensitivity factors (RSF) on homogeneous samples of the known composition [33]: Si_75_Ge_25_ and Si_68_Ge_32_. Ge concentration in the said cases under the standard RSF was 19 and 25 at.%, respectively. In the both cases, calibration coefficients close in values were obtained: for the first sample, it is *k*_*1*_ = 0.76 and for the second one. –*k*_*2*_ *=* 0.78, respectively.

**Fig. 4:**
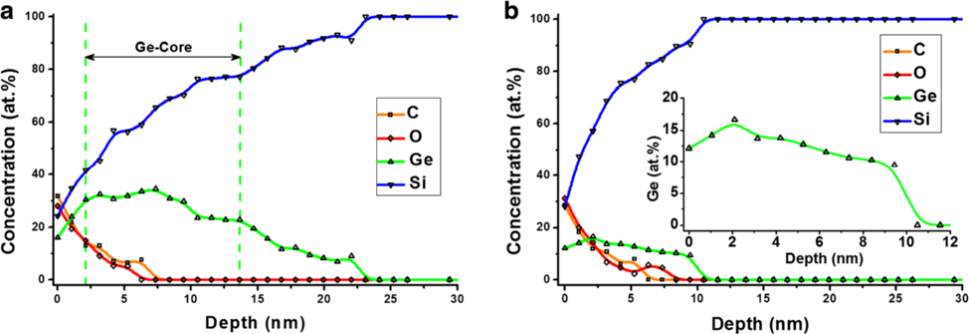
Concentration depth profiles of a single p-cluster and wetting layer. Concentration depth profiles of a single p-cluster (**a**) and wetting layer (**b**) recorded on the surface of specimen А 2 with the buffer layer Si_0.9_Ge_0.1_ 10 nm thick. The scaled-up beginning part of the Ge wetting layer concentration depth profile is shown at the *inset* (**b**)

The concentration depth profiles of a single p-cluster and the wetting layer on the surface of the specimen А 2 with the buffer layer Si_0.9_Ge_0.1_ 10 nm thick was obtained. They are presented in Fig. 4a, b, respectively. Sample A 2 was selected to construct the profiles, because it has a buffer layer of the known geometry and composition. Section of the profile corresponding to the buffer layer can be used to check the accuracy of the composition calculation and determining the rate of ion etching, as well as to identify the effects of ion etching and evaluate depth resolution when profiling.

However, it turned out that, unlike the other samples studied in the paper, its surface layer having a thickness of about 5 nm, contains appreciable amounts of C and O. We assume that C and O came to be in the sample during its keeping and not during its manufacturing. The reason for such a scenario, on one hand, is the same penetration depth of C and O in a p-cluster (a) and buffer layer (b). On the other hand, the same scenario indicates the fact that carbon and oxygen is absent in the buffer layer below the p-cluster (a).

Indeed, clean surfaces in contact with the air atmosphere adsorb C and O. The atoms of C and O can penetrate into the sample for appreciable distance even at room temperature and a short storage time, as they are interstitial impurities in the lattices of Si and Ge [40]. The diffusion coefficient of interstitial impurities is several orders of magnitude higher than the diffusion coefficient of substitutional impurities [40].

Initial surface of the specimen before ion etching corresponds to zero depth on the depth profile. Thus, it contains maximal quantity of carbon and oxygen. With layer-by-layer removing the specimen surface, the C and O quantities decrease and reach zero level at the depth ≈ 6 nm (Fig. 4).

Type of Ge distribution in the depth profile of the wetting layer almost duplicates the buffer layer geometry (Fig. 4b). Such behavior of Ge distribution testifies to the fact that there is no wetting layer in this specimen because p-clusters growth began on the buffer layer.

The determined Ge content in the initial section of the depth profile (0–6 nm) has an overestimated value, and it corresponds to the technological composition of the specimen buffer layer 10 at.% in the final section of the depth profile (7–10 nm). In our opinion, the Ge content overestimated value in the buffer layer initial section is caused by the presence of C and O in it. Indeed, we performed the RSF calibration on Ge/Si-alloys that do not contain C and O and got the correct values for Ge and Si content in the buffer layer final section. However, RSF in the pure Ge/Si-system differs from their values in the C/O/Ge/Si-system. Therefore, their use in the presence of C and O overestimates Ge content and underestimates Si content. It follows from above that the final section of the p-cluster depth profile (7–30 nm in Fig. 4a) has the correct value of Ge and Si contents, and its initial section has overestimated Ge content and underestimated Si content.

Agreement of step length on Ge depth profile of the wetting layer with buffer layer thickness (see inset in Fig. 4b) indicates that we correctly determined the ion etching rate, while its sharp right edge shows that the chosen ion etching mode was not accompanied by the intensive processes of atoms mixing under ion etching. The same sharp decrease of Ge may be observed on the right edge of the depth profile of p-cluster (a). This is because the p-cluster base is on the buffer layer.

The sharp transition from the buffer layer to the Si-substrate on the wetting layer depth profile (b), which occurs during a single ion etching step, indicates that the magnitude of depth profiling resolution does not exceed its length, i.e., is not more than 1 nm. This assertion is consistent with the fact that the escape depth of the Auger electrons of Ge and Si is less than 1 nm [33]. In other words, when profiling the substrate effect may begin to appear at the approach to it at a distance closer than 1 nm or, respectively, one ion etching cycle.

The type of Ge depth distribution in p-cluster (a) is of no surprise. Obviously, it is conditioned by the Si and Ge interdiffusion process, whose traces are clearly seen on the Auger maps of germanium and silicon (Fig. 2). The depth profile of p-cluster is specified by the following factors: we pass SiC_x_O_y_-enriched shell of the p-cluster in the first 3 nm (Fig. 4a) and penetrate into the Ge core, where Ge quantity at the depth of 5–6 nm reaches its maximum value of 34.5 at.%. Drop of Ge content with moving deep further is conditioned by the processes of its diffusion into the substrate.

It is known that diffusion of Si atoms in a p-cluster and a wetting layer leads to a significant relaxation of their stress states [36]. We believe that carbon and oxygen for the same reason extensively diffuse into the p-cluster and the buffer layer. They replace their silicon, since they play the same role with it. Therefore, in this case, instead of the Si-enriched shell, we have the SiC_x_O_y_-enriched shell surrounding the Ge core of the p-cluster (a).

**Fig. 5:**
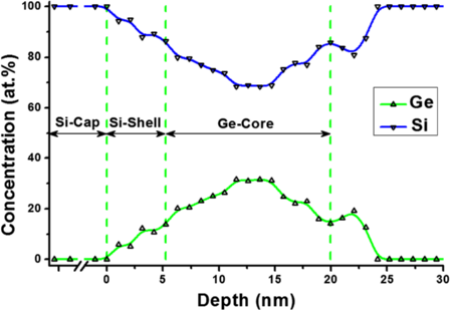
Concentration depth profile of d-cluster. Concentration depth profile of d-cluster registered on B 1 sample, which begins with Si-cap layer and stretches across Si-shell, Ge-core up to Si-substrate

In order to study the nanoisland near-surface layers composition not redistributed under the influence of the C and O penetration in them, B 1 sample was taken. In this sample, the nanoislands array immediately after growing at 700 °C was covered with Si-cap at a temperature of 300 °C. The relatively low temperature of capping provided suppression of diffusion processes between the nanoislands array and the cap layer. Figure 5 shows the concentration depth profile of a capped d-cluster of the sample. In the presented concentration profile, one can clearly see in sequence from left to right three zones: the Si-cap layer, Si-shell penetrating into nanoisland to a depth of 5–7 nm, and Ge-core.

If for the buffer layer (Fig. 4b), we take the ratio of the sum of C, O, and Si concentrations to the Ge concentration, its value will be close to the nominal ratio value, into which, it turns with the disappearance of C and O with depth. In other words, the buffer layer depth profile contains no obvious signs of Si and Ge interdiffusion. Small concentration gradients and low stresses in the buffer layer-substrate interface area do not promote the development of interdiffusion processes.

### 3D Composition Distribution in Single Nanoisland

With developed procedures for determination of the lateral (Auger map) and depth (concentration depth profile) distributions of the elemental composition in single nanoisland, it is easy to obtain 3D composition distribution within its bulk [41]. It is only needed to get the lateral distribution of the elemental composition in the nanoisland while recording the concentration depth profile at several depths and then interpolate the obtained data by the nanoisland bulk.

**Fig. 6:**
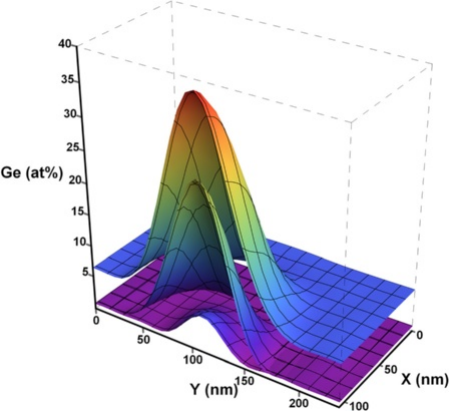
Three Auger maps in 3D representation for Ge content distributions. Fragments of the surfaces of three Auger maps in 3D representation for Ge content distributions recorded in d-cluster at the depths of 8, 17, and 27 nm, correspondingly

**Fig. 7:**
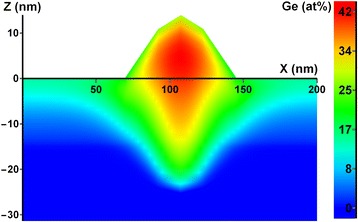
Axial section of 3D distribution of Ge content in d-cluster bulk

Let us illustrate the indicated procedure using d-cluster on the A 1 specimen as an example. In the case under consideration while obtaining concentration depth profile, there were recorded three lateral distributions of d-cluster composition at the depths of 8, 17, and 27 nm, correspondingly. They are shown in 3D representation for Ge in Fig. 6. This figure is an impressive illustration of the fact that there is a germanium core at all depths in d-cluster, which becomes less pronounced while moving depthward and even penetrates into the substrate. It should be noted that the lateral distribution of Ge recorded at the depth of 27 nm (low 3D surface in Fig. 6) corresponds to a level under the wetting layer-substrate interface. Ge content distribution by d-cluster bulk was calculated from the presented data by the approach of polynomial approximation. Its axial section is shown in Fig. 7 where one can vividly see the germanium core in the nanoisland and its shape.

It is known that d-cluster has regular-shaped faceting as p-cluster with inclination angle of the side facet to the base 10.9° on the “pedestal” as a truncated pyramid with inclination angle of the side facet 26.6° [39]. Knowing the total height of the d-cluster, it is easy to obtain its axial section geometry from the data of the concentration depth profiling (difference in the depths of the concentration profiles of d-cluster and the wetting layer) and two indicated angles. The height of the dome defined by the above method is in good agreement with the data obtained for this sample by AFM. Thus, the dome, shown in Fig. 7, has an estimated height of 13.75 nm, which corresponds to the center of the dome height distribution shown in Fig. 3.

### Outline of the Ion Etching Geometry and the Formed Crater

Ion etching technique may be also used for opening inner layers of heterostructures for their study. It should be noted though that often the data on the geometry of heterostructures are not accurate in such cases. It is most easy way to determine accurate geometry of heterostructure by obtaining its cross section.

**Fig. 8:**
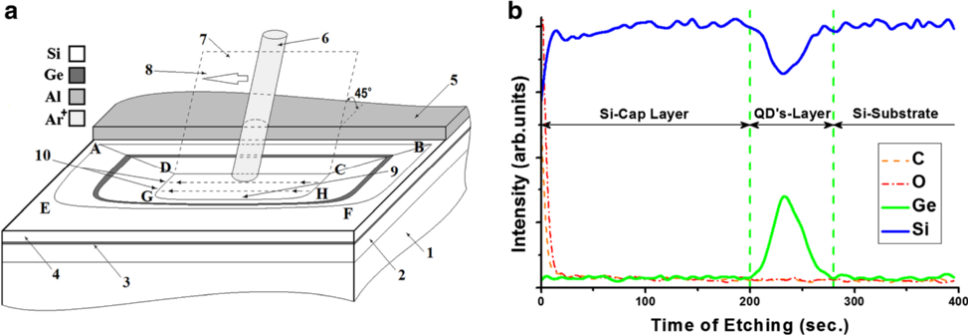
Outline of ion etching crater geometry (**a**) and concentration depth profiles of B 1 specimen (**b**). (**a**) Outline of ion etching geometry and the formed crater: 1 – Si substrate, 2 – Si buffer layer, 3 – GeSi QD’s layer, 4 – Si cap layer, 5 – Al foil shield, 6 – Ar^+^ ion beam, 7 – ion beam scanning plane, 8 – ion beam scanning direction, 9 – etching crater bottom, 10 – ion beam scanning lines; (**b**) Concentration profiles of depth distribution of elements C, O, Ge and Si in the process of ion etching on B 1 specimen

Figure 8а shows outline of the ion etching geometry, where ion beam 6 is moving on the surface of heterostructure on the 1 × 1 mm raster, whose part is covered by the aluminium shield with a smooth edge 5 used for obtaining ABCD cross section of the heterostructure at the preset angle of 45°. The ion beam is moving in the scanning plane 7 inclined at an angle of 45° to the specimen surface in direction 8. There is parallel shift of plane 7 by scanning lines 10 when moving by raster. Crater with a plane bottom 9 and ABFEDCHG lateral surface forms on the surface of the heterostructure in the course of ion etching. This is a cross section of the heterostructure.

**Fig. 9:**
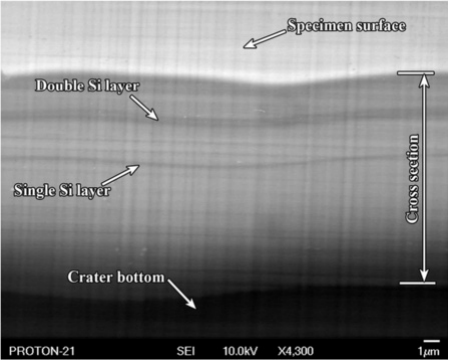
SEM image of the multilayer film cross section. SEM image of the cross section of the multilayer film coating with inclination angle to the crater bottom plane 45^0^ on B 2 specimen

A concentration depth profile shown in Fig. 8b is recorded in the process of etching on B 1 specimen. It is possible to determine accurate geometry of the heterostructure by its cross section ABCD (see Fig. 8а), whose scanning image fragment is shown in Fig. 9. In this case, heterostructure layers may be identified by using the concentration depth profile (Fig. 8b).

Analyzing Fig. 8, it is easy to understand that Si cap layer 4 (а) corresponds to the initial section of the concentration profile from 0 to 200 s (b), GeSi QDs layer 3 (а)—to the section from 200 to 280 s (b), and Si buffer layer 2 and substrate 1 (a)—to the section after 280 s of etching (b).

After determining accurate geometry of the heterostructure and identifying its layers by the ion etching, it is possible to open its any layer of interest for the study. For example, for opening a layer of QDs 3 in the considered B 1 specimen, it is necessary to move to a new site of the sample surface and perform etching under the same mode during 200 s. After opening the layer of interest, it becomes possible to obtain both lateral distribution of the composition in the QDs and to get their concentration depth profiles.

**Fig. 10:**
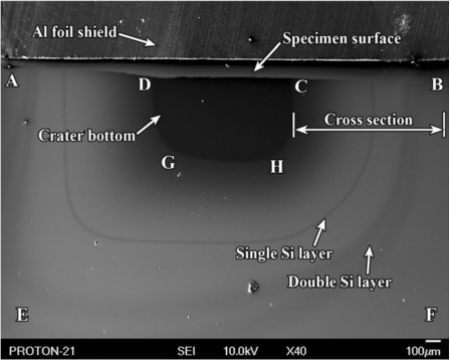
SEM image of the formed crater. SEM image of the formed crater of the multilayer film GeSi/Si structure resulted from ion etching on B 2 specimen

Figure 10 shows a scanning secondary electron image of the typical ion etching crater of heterostructure obtained by the above described procedure on B 2 specimen. Here, the cross section of the heterostructure at the preset angle of 45°, as in the outline of the ion etching crater (see Fig. 8а), is represented by ABCD reverse trapezoid. Single and double Si-enriched layers in SiGe alloy can hardly be identified on the scanning image of this cross section fragment (Fig. 9). The double silicon layer contains GeSi QDs inside. It is not convenient to use this section for study of GeSi QDs because of close location of silicon layers to each other. However, this cross section is important as it allows determining accurate geometric parameters of the studied heterostructure by the known inclination angle.

Lateral surface of the crater consists of two parts: its ABCD section is a plane formed due to sputtering of the heterostructure material by the primary ion beam with fixed geometry, while bent lateral surface ADGHCBFE (see Figs. 8а and 10)—due to sputtering of heterostructure material by the backscattered ions of the primary beam. Inclination angle of the bent lateral surface to the plane of the crater bottom is not the same in various sites but in any site, it does not exceed 1°. This secant surface due to its geometrical enlargement is more convenient for study of the closed located thin layers of the heterostructures.

Really, if double Si layer on ABCD secant surface is hardly seen even at ×4300 magnification (Fig. 9), then both layers of the double Si layer (Fig. 10) are well resolved and seen at magnification ×40 on the small angle cross-section surface.

**Fig. 11:**
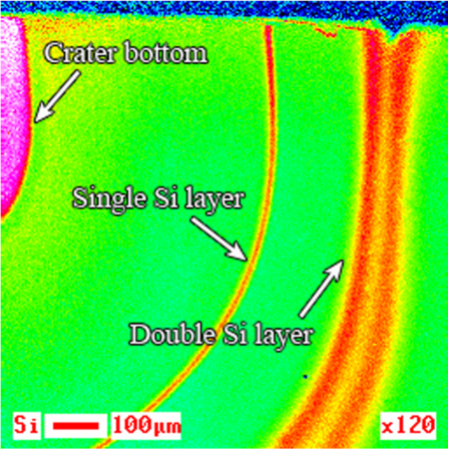
Auger map of Si distribution in the small angle cross section. Auger map of Si distribution in the small angle cross section of multilayer film coating on B 2 specimen

Figure 11 shows the Auger map of Si distribution recorded on the area of CBFH crater lateral surface (Fig. 10) of the heterostructure small angle cross section on B 2 specimen. Dark lines in Fig. 10 correspond to the Si-enriched layers in the Auger map (Fig. 11). There are GeSi QDs inside Si double layer, while an area with maximal Si content corresponds to the crater bottom.

### Small Angle Cross Section of QDs Layer in Capped Heterostructures

**Fig. 12:**
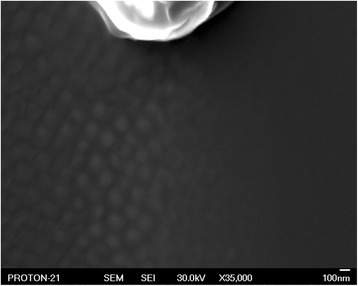
SEM image of small angle cross section. SEM image of small angle cross section surface of the layer containing GeSi QDs on B 1 specimen

Let us illustrate the possibilities offered by the small angle cross section of heterostructure on the lateral surface of the crater for studying distribution of its inner layers composition on B 1 specimen. Figure 12 shows a fragment of ADGE crater lateral surface (see Figs. 8 and 10) containing a layer of buried GeSi QDs. The fragment areas located on the right correspond to the greater depth of occurrence in the initial heterostructure than the areas located on the left. In the central part of Fig. 12, there is a layer of QDs cut at different heights from their top to their bottom. Its left part is a transition between Si cap layer and GeSi QDs, while the right part is a transition between GeSi QDs and buffer layer, respectively.

Data about the elemental composition distribution in all three areas is very important for understanding of both—mechanisms of GeSi QDs nucleation and physical processes taking place while they are growing and covering with the Si cap layer.

**Fig. 13:**
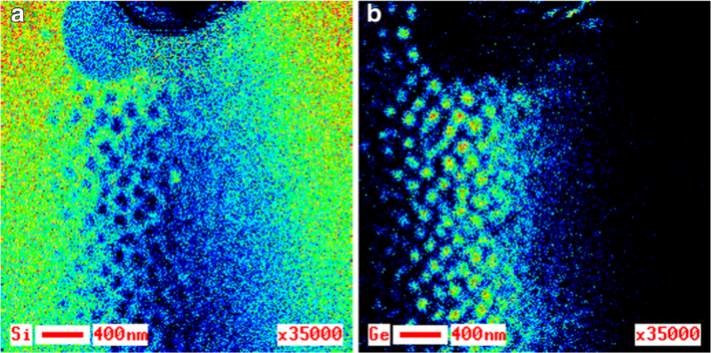
Si and Ge Auger electron maps. Auger electron maps of Si (**a**) and Ge (**b**) distributions on the surface of small angle cross section of the layer containing GeSi QDs on B 1 specimen

Figure 13 shows the distributions of Si (a) and Ge (b) on the lateral surface fragment of the crater containing a layer of QDs and presented in Fig. 12. Ge distribution is more informative here. For example, it is well seen that QDs contain the germanium core in all their cross sections. Moving by the Ge Auger map from left to right, one can observe both its lateral distribution in QDs at different depths and in transitional layers adjacent to them from above and from below. In fact, the Ge Auger map contains all data, which we obtained for constructing 3D distribution of composition in d-cluster when recording of lateral distribution of elements in its cross sections and its concentration depth profile.

**Fig. 14:**
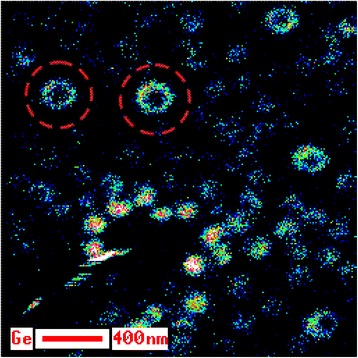
Ge Auger electron map of the small angle cross section surface. Auger electron map of Ge distribution on the surface of small angle cross section of the layer containing GeSi QDs on B 1 specimen. The rings enriched with Ge (warmer color area) are marked with dotted red circles

**Fig. 15:**
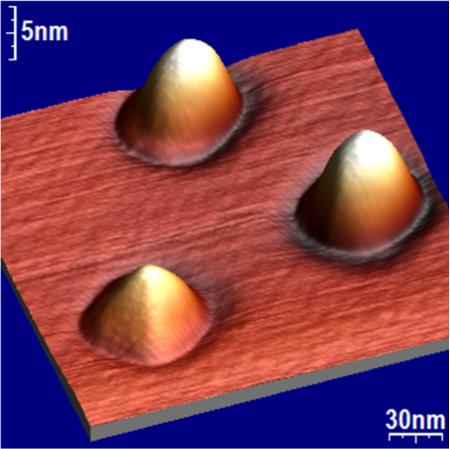
The grooves around the bases of GeSi-nanoislands registered by AFM on A 1 specimen

In order to learn in a greater detail, Ge distribution in the transition layer between QDs bases, wetting and buffer layers, we recorded one more Auger map of Ge distribution for the indicated area of cross-section surface at large magnification (see Fig. 14). Here, one can identify rings enriched with germanium (warmer color area marked with dotted red circles in Fig. 14). The rings were found on the section of the sample, which was formed as result of etching by a backscattered ions beam incident at a small angle (look at ADGHCBFE section on Figs. 8a and 10). Their formation, in our opinion, is not associated with the occurrence of the surface relief by ion etching, as the etching by ion beam at small angles to the surface does not contribute to its occurrence. In our opinion, they form as a result of Ge intensive diffusion into the substrate on the GeSi QDs lateral surfaces in the processes of their evolution and growth. The indicated rings are located under the grooves surrounding the QDs bases (see Fig. 15) [28].

Let us indicate advantages for study provided by the small angle cross section of heterostructures due to geometrical enlargement. According to our estimation, width of the layer on the small angle cross section more than 60 times exceeds its width on the vertical cross section. For example, width of the GeSi QDs layer on the small angle cross section shown in Fig. 13 reaches 1.4 μm, while real thickness of this layer in the studied heterostructure is 24 nm.

## Conclusions

We have shown that application of SAM with ion etching technique in combination with the effective correction of the thermal drift of the studied object analyzed area allows direct local study of composition distribution in the bulk of single Ge_x_Si_1 − x_ nanoislands both in open and capped nanostructures with lateral size of the structure element down to 8–10 nm. Lateral distributions of the elemental composition and concentration depth profiles of single GeSi/Si nanoislands in the open nanostructures were recorded. 3D distribution of the chemical composition in the d-cluster was obtained by several lateral composition distributions and concentration depth profile by the interpolation approach.

It was shown that the nanoislands of both types contain germanium core at all depths, which becomes less pronounced while moving depthward and even penetrates the substrate. In studied nanostructures, maximal Ge content may reach a level of about 40 at.%. The Ge core in nanoislands is surrounded by a Si-enriched shell. Diffusion along the lateral surface of GeSi nanoislands should play a key role in the formation of such structure [42]. C and O atoms adsorbed on the surface of the heterostructure, can penetrate several nanometers into the Si-enriched shell and wetting layer. This process leads to a significant relaxation of their stress states. In this case, instead of the Si-enriched shell, we have the SiC_x_O_y_-enriched shell surrounding the Ge core of GeSi nanoisland.

The authors demonstrated a procedure for determining accurate geometric parameters of multilayer planar heterostructures by obtaining their cross sections by means of ion etching technique and a procedure for precision opening of any of their layers with the aim of its study (see Figs. 8, 9, 10, and 11). We showed great possibilities for study offered owing to geometrical enlargement by the heterostructure small angle cross section located on the lateral surface of the ion etching crater and formed due to sputtering its material by the backscattered ions of the primary beam. The small angle cross section allows observing both lateral distribution of Ge in QDs at different depths and in the transition layers adjacent to them from above and from below. Ge-enriched rings located under the nanoislands and spread from the wetting layer deep to the substrate are discovered. They are located under the grooves surrounding QDs bases. They are formed because of intensive Ge diffusion into the substrate by the GeSi QDs lateral surfaces in the process of their evolution and growth.

In the author’s opinion, such GeSi-nanoislands structure features observed in the study as the Si-shell, Ge-core penetrating the substrate, the grooves surrounding GeSi-nanoislands bases and Ge-enriched ring-shaped regions located in the substrate under the grooves are an obvious result of the completeness of the interdiffusion processes course in the nanoisland-substrate system. They point us in the direction of the evolution of the structural state of the system when it is tending to the equilibrium state. However, the way that the system can go in this direction, obviously, depends on the conditions in which it was in the process of its manufacture. In other words, not all of the mentioned structural features of the GeSi-nanoislands can be observed in conditions not conducive to the development of diffusion processes (short-time intervals and low temperatures of growth and high deposition rates).

## Abbreviations

AFM: atomic force microscopy
LED: light-emitting diode
MBE: molecular beam epitaxy
QDs: quantum dots
RSF: relative sensitivity factors
SAM: scanning auger microscopy
SEM: scanning electron microscopy
STM: scanning tunneling microscopy
TEM: transmission electron microscopy
